# Transitions from pediatric to adult IBD care: Incorporating lessons from psychogastroenterology

**DOI:** 10.3389/fgstr.2022.1037421

**Published:** 2022-10-12

**Authors:** Michelle L. Mendiolaza, Jordyn H. Feingold, Halley P. Kaye-Kauderer, Marla C. Dubinsky, Ksenia O. Gorbenko, Laurie A. Keefer

**Affiliations:** ^1^ The Susan and Leonard Feinstein Mount Sinai Inflammatory Bowel Disease Center, Department of Pediatrics, Icahn School of Medicine at Mount Sinai, New York, NY, United States; ^2^ Department of Medical Education, Icahn School of Medicine at Mount Sinai, New York, NY, United States; ^3^ Department of Population Health Science and Policy, Icahn School of Medicine at Mount Sinai, New York, NY, United States; ^4^ The Susan and Leonard Feinstein Mount Sinai Inflammatory Bowel Disease Center, Department of Gastroenterology, Icahn School of Medicine at Mount Sinai, New York, NY, United States

**Keywords:** adolescents, inflammatory bowel disease, gastroenterology, psychosocial, pediatrics, transitions, patient-related interventions, mental well being

## Abstract

The transition from pediatric to adult gastroenterology care for adolescents with inflammatory bowel disease (IBD) is a critical period associated with poor disease outcomes and high medical costs. Burdens such as the discontinuity of care when transitioning from one provider to another are amplified by poor coping and psychosocial factors. However, existing research on the topic of health care transitions has centered largely on disease knowledge and competencies that young adults ought to master and self-manage, while largely disregarding the broader psychosocial context and impacts of IBD on daily functioning. Findings from a recent mixed-methods pilot study of transition-aged adolescents with IBD and their parents highlight the importance of acknowledging the psychosocial needs of adolescents with IBD and their families throughout the transition process, which include understanding the gut-brain axis, optimizing social support and mental health resources, and maintaining optimism and positivity. In this review, we expand upon the findings from this pilot study, synthesize the latest research in psychogastroenterology and pediatric-to-adult transitions in IBD, and provide five patient-centered interventions that may be implemented in clinical settings, in anticipation of, and during the patient transition experience. These interventions are rooted in positive psychology and cognitive-behavioral principles and are designed for adolescents with IBD to complete with input from their families and health care professionals.

## Introduction

### IBD and its psychosocial implications for children and young adults

Inflammatory bowel diseases (IBDs), which include Crohn’s disease and ulcerative colitis, are chronic disorders of the gastrointestinal (GI) tract with systemic manifestations that are frequently diagnosed in childhood and adolescence (1). Pediatric-onset disease can be especially unpredictable and stigmatized, interfering with several aspects of development and social functioning (2). Research shows that symptom severity (e.g., abdominal pain and fatigue) and disease activity (e.g., gastrointestinal inflammation)—which are not always correlated due to high rates of superimposed irritable bowel syndrome (IBS)-like symptoms—co-determine the overall burden of IBD. The patient’s ability to cope adequately with their symptoms is another critical factor in reducing disease burden (3).

Pediatric IBD cannot be disentangled from its psychosocial context. Children and adolescents with IBD may feel ashamed by fecal incontinence and experience social anxiety due to school absences, challenges around eating, and frequent bathroom visits (4). Additional factors such as age, social support, and degree of stress may also influence how adolescents manage and react to their IBD (4). These factors can contribute to a significant psychological burden that influences disease outcomes and patient identity (5).

Increasingly, IBD is being recognized as a complex disorder that can be studied through the lens of *psychogastroenterology* (6). Several studies have found that adolescents with IBD are at greater risk for developing major depressive disorder than age-matched individuals in the general population, and even those with other chronic diseases (7, 8). Rates of depression are as high as 25% within the adolescent IBD population and studies in pediatric and adult populations show a significant relationship between depression and anxiety with IBD disease activity (9, 10). One could imagine that mental health comorbidity might be a direct result of the burdens of living with this chronic disease. Research also shows that the relationship between IBD and mental illness is bidirectional, unified by systemic inflammation that inflicts damage on the body’s immune and nervous systems (11–13). Finally, patients with IBD and co-morbid depression and/or anxiety may be less likely to adhere to standard medical care (14, 15).

A large body of research has recognized these complex relationships, and the promise of psychological and behavioral interventions to treat symptoms and improve quality of life in patients with IBD (6). However, a dearth of research exists specifically studying the applications of psychogastroenterology principles during transitions from pediatric to adult GI care.

### Transitions from pediatric to adult IBD care: A particularly vulnerable time

It is well-understood that disruptions in care as patients with IBD undergo pediatric-to-adult transitions can result in poor health outcomes and high medical costs (16, 17). As patients with pediatric IBD progress into adulthood, the chances of disease-related complications increase, and burdens of IBD are amplified by psychosocial factors including poor coping ability. Notably, 15% of patients with IBD account for roughly 50% of health care costs, many of whom experience concomitant chronic pain, depression, and poor social support (18, 19).

Ideally the transition from pediatric to adult care takes place when young adults have achieved some degree of disease knowledge, including an understanding of treatment plans and the ability to gather critical information and self-advocate to health care providers (20). Young adults should also be in relatively stable health and have sufficient self-efficacy and enthusiasm for active communication regarding self-care behaviors (21). Clearly, these higher-order cognitive and self-management skills may be undermined in the setting of emerging mental illness or other psychosocial problems.

A study comparing health priorities among adolescents with IBD and their physicians found significant differences between patient and physician priorities. While physicians focused on getting their patients to achieve clinical remission and avoid surgery, adolescents were more concerned with social aspects such as going to school, dating, and fitting in with peers in their age cohort (22, 23).

To date, there remain no evidence-based guidelines on the timing of initiation and completion of transitions, or the specific competencies required of transitioning young adults. Consequently, the ability of physicians to guide the patient in transition preparedness varies greatly across settings. Roughly 80% of adult gastroenterologists report inadequate preparation of patients transferred to their care from the pediatric setting (20). There is also a knowledge gap for adult gastroenterologists, with many believing that they themselves are not sufficiently trained in adolescent medicine topics to provide appropriate care to newly transitioned adolescents (24). Research in this arena is warranted to determine best practices for transitions and how to support patients and their families to optimize transition-readiness.

Whereas most of the existing transitions literature centers primarily around logistics and the mechanics of disease management, further investigation is needed to thoroughly examine the role of psychosocial factors in altering one’s ability to successfully transition. To address this gap, our team conducted a single-center pilot study to explicitly examine the impact of psychosocial factors on transitions from the perspectives of both adolescents with IBD and their parents. The results of this study, described below, suggest that attention to psychological and social factors are integral in understanding the transition experience of patients and families.

## Findings from a recent pilot study that aimed to understand transitions from a psychosocial perspective

In a single-site, qualitative pilot study utilizing the method photovoice (25), a community-based participatory research method aimed at empowering research participants to share their perspectives through photographs and semi-structured interviews, we recruited adolescent participants (aged 14-23) diagnosed with IBD and their parents to share their experiences living with IBD, centering around the pediatric-to-adult care transition (26). Both adolescents living with IBD and their parents took photographs and drafted captions in response to prompts such as, “what does it mean to be a pediatric vs. an adult patient” (for parents, “what does it mean to be the parent of a pediatric vs. an adult patient?”). Participants submitted these photos and sat for interviews with the research team to discuss the photos and elaborate on their experiences. Interviews were recorded, transcribed, and analyzed for themes using MAXQDA Analytics Pro 2020 (Version 20.0.7, Berlin).

The key findings from the pilot study are outlined in **Table 1**. In summary, participants discussed barriers to transitions including psychological distress, disease uncertainty, gut-brain axis-related issues, a lack of understanding by people unaffected by IBD, and frequent life disruptions. Facilitators of transitions included having a disease narrative, deliberately shifting responsibility for disease management tasks, possessing positivity and optimism, adequate social support, engagement with the IBD community, and mental health support. A sampling of quotes from adolescents regarding their transition experiences is outlined in **Table 2**.

**Table 1:** Results from the pilot study.

Transition Barriers Identified	Transition Facilitators Identified
*Psychological distress* (e.g., body image concerns, depression, anxiety, fear of the future) *Disease uncertainty and lack of control* (i.e., not knowing when IBD would act up, challenges around socializing, eating, using public bathrooms) *The gut-brain axis* (i.e., whether symptoms are due to stress or the mind or a result of ‘real’ inflammation) *A lack of understanding by others* who are not impacted by IBD *Navigating the many disruptions to daily life* (e.g., missing school or social engagements)	*The transfer of disease management skills* from parents to children (with support and two-way communication, consistent with prior transitions literature) *Possessing a disease narrative* with which to talk about IBD with others *Maintaining optimism and positivity* (i.e., a belief that things will get better, that having IBD has imparted valuable lessons of strength and resilience) *Being embedded in an IBD community* (e.g., through volunteering, participating in support groups, Camp Oasis) *Having adequate social support* (from friends and family) *Mental health support* (i.e., talking to a therapist or health psychologist)

**Table 2:** Barriers and facilitators for transitions.

Barriers for Transitions	Corresponding Quotes from Adolescent Subjects	Facilitators for Transitions	Corresponding Quotes from Adolescent Subjects
*Topic*	*Quote*	*Topic*	*Quote*
**Psychological Distress**	“I don’t want to believe that life will never return … I always want to say, ‘the light’s going to come back. It’s not all bad. Sometimes it has to get worse before it gets better’… I just fear that I can dive into that pit and never come out.”	**Disease Self-Management**	“[My parents] really empowered me to know that I could and should take care of myself, but that I would always have them as a safety net … In college I definitely relied on them quite a bit in terms of ordering my medication and handling so much of the logistics.”
**Disease Uncertainty & Lack of Control**	“For me there was a lot of uncertainty and I feel like my IBD has just made a lot of things unpredictable and that can be scary because I don’t know what the future holds. I don’t know how long the medication will work or how long until, you know, something bad could happen. And I tried to not think about those things, but obviously there’s always that uncertainty…”	**Disease Narrative**	“I was at a support group once and they were asking for suggestions about how to talk to your friends about [IBD]. What I told them is that … at least initially from a very mechanical sort of place, like ‘yeah, I have Crohn’s disease. It means that my immune system is attacking the inner lining of my colon. It causes so much pain and other symptoms’… Not in a pity like, ‘Oh yeah, like my stomach aches sometimes.’ Not to say that I don’t just straight up complain sometimes, but … the first time that you’re talking about it to set the tone and sort of very matter of fact like ‘it is what it is’ I find that that has really helped me want to talk about it … Again, it’s just about setting the tone about how you want others to engage by just doing it yourself.”
**The Brain-Gut Axis**	“I know not all of the pain I’m having is caused directly by Crohn’s, a lot of it could just be in my mind, and it bothers me that I can’t separate that … Often when [my pediatric GI] asks how my pain is … what is from stress of school and things going on at home and what is actually from the Crohn’s, I just don’t know how to differentiate between the two … I’m afraid of what my mind can do to myself.”	**Optimism and Positivity**	“I think positivity is the best way to get through any tough time and everybody that has Crohn’s has a different experience, but as long as you stay positive and enjoy the good parts of your life, you’ll have good memories to look back on.”
**Others Don’t Understand**	“You know, there are people that are good and completely understand it, and I’m like, ‘it’s just been a bad day,’ they’re like, “stay home, it’s fine.” And then, there’s other people that just don’t get it. And I think … the biggest problem I have is the fatigue and the body aches and the muscle aches just like all the time. Just going through a regular day and I don’t get enough sleep like my whole body hurts and it’s just like a whole ‘nother level of exhaustion. And people don’t really understand that.”	**The IBD Community**	“I do have a lot of friends with IBD because I’m on the Crohn’s and Colitis Foundation’s National Council of College Leaders … so I’ve met literally dozens and dozens of people my age with IBD … I know if I’m ever having a problem, I can message literally any of them and talk to them. I also have a therapist that I see. So I have a variety of places I can go depending on what I want and need help like coping with.”
**Disruptions to Daily Life**	“Last week, I spent a third of the day just sitting in the nurse’s office because I was in so much pain from eating. I couldn’t lay down because that hurt more, so literally just sitting there … I was sweating from the pain … So, the next day I was like, no … not going to school.”	**Social Support**	“Once I kind of grew up a little bit and started taking on more, I was like, ‘okay, like I need to do whatever it takes to be at the health that I want. And that includes reaching out to ask for help…’ And, and realizing that doesn’t make me any less adult or um, you know, self-sufficient … Especially when I’m across the country [my mom] sends me reminder messages or just checks in on me. And you know, I am good about it, I don’t have too much pride to not ask her when I need help because you know, everyone needs help.”
		**Mental Health Support**	“Talking with [my health psychologist] … helped me understand that what I’m dealing with is chronic … I never really understood what it actually meant to have an illness that’s not going anywhere … I would tell people to reach out to a social worker or a psychologist to get that rundown.”

The findings from this study support the idea that transition needs and transition programs should go beyond individual factors such as improving patient knowledge or self-management skills, and place attention to the social context, such as psychological well-being and community factors, that influence disease outcomes (27).

In the next section, we highlight opportunities for adolescents and their families to engage in to help improve transition-readiness. We propose five tangible interventions that stem directly from the findings of the pilot study. These interventions are customizable and can be adapted for the specific needs of each individual patient. Further, these can be tested in existing or novel pediatric-to-adult IBD transition readiness programs.

## A model for testing—Five interventions for facilitating transitions

The following are recommendations that can be implemented in the pediatric IBD care setting or as part of workshops for transition preparedness. The interventions stem directly from the pilot study data and emphasize the psychosocial dimensions of pediatric-to-adult transitions. Further, they are rooted in positive psychology and cognitive-behavioral principles, which may be associated with improved disease outcomes across the spectrum of digestive disorders (28, 29). While we present a variety of suggestions for use, additional research is needed to evaluate the impact of these exercises as discrete interventions to be incorporated in psychosocial programs.

Finally, these interventions can be introduced to patients at any age and health care professionals need not wait for the transition to suggest these. However, if not previously initiated, the pediatric-to-adult transition may be an impetus to offer these interventions.

### Develop a disease narrative

Developing a disease narrative with which to explain IBD to others emerged as a protective factor in the pilot study. Such narratives highlight ways to talk about IBD with others who may not know about the disease. Prior to transitioning, pediatric patients may benefit from establishing their own disease narrative, akin to an *elevator pitch*, for curious friends and family members, or in social situations when meeting new people.

Elements of a disease narrative might include:

1. What is IBD and what symptoms are most prominent for you day-to-day?
2. What are some of the challenges that you might experience related to having IBD (e.g., managing medications, medical procedures, dietary or activity restrictions)?
3. In what ways have you grown through the challenges of living with IBD?

Discussing how the disease has shaped the patient’s lived experiences in a positive way can challenge patients to adopt an *optimistic explanatory style.* This way of communicating, which emphasizes areas of growth and learning through challenges, is associated with happiness, gratefulness, and lower levels of stress and depression (30).

This exercise can be encouraged by pediatric doctors well in anticipation of transitions to adult care. This is a way for patients to build self-efficacy, take ownership of their own medical histories, and advocate for themselves when they reach adult-centered care.

### Practice gratitude

Embracing optimism and positivity, as well as having adequate social support, are critical in helping the transitioning patient. Explicitly discussing gratitude is a way for patients to not only reflect on existing resources and social support, but also to enhance support and strengthen existing connections.

Patients may be encouraged to develop a gratitude practice, reflecting on the following ideas:

- What is something you are grateful for today?
- Who in your life is there for you to help you cope with and manage your IBD?
- Who have you been able to share thoughts or ideas with?

*Gratitude Activity 1: Write a gratitude letter*

Consider writing a gratitude letter to someone who has helped you in some way. It is okay if you have not spoken to this person in a long time. Spend some time expressing how they have helped you and why you are thinking of them now.

*Gratitude Activity 2: Keep a gratitude journal*

Many patients with IBD keep symptom logs or diaries. We strongly encourage this through the transition period. Even through occurrences of high disease activity, patients can record a few moments in their lives for which they feel grateful on a given day. These may be related to IBD or not (e.g., “my professor was very understanding and sent me a long email with everything I missed from class when I had to leave due to my fatigue” or “I am grateful that I no longer have to go to the health center for my meditations, Humira has made it easier to navigate meds in college”). Routine gratitude practice can subserve overall well-being and help individuals attune to the positives in life, even when days are challenging.

### Pay it forward

Engagement with the IBD community through volunteering and mentorship was a powerful source of strength for adolescents in our pilot study. As adolescents prepare to take on an adult patient identity, one way to bolster their self-efficacy is for them to invest time within the IBD community (e.g., by mentoring patients recently diagnosed with IBD). Indeed, helping others through acts of kindness or philanthropy is associated with increased happiness and life satisfaction (31).

A great place to start is through the Crohn’s and Colitis Foundation of America Chapter (CCFA), and a provider or caregiver can help the patient find their local chapter: https://www.crohnscolitisfoundation.org/chapters. Opportunities include serving as a Power of Two Mentor, getting involved in Camp Oasis, participating in a Take Steps Walk, and facilitating a support group.

### Set SMART goals to transfer disease-management tasks

Certainly, a huge part of transitioning to adult care is the deliberate transfer of disease-management tasks that were previously handled by caregivers including symptom tracking, medication management, meal preparation, scheduling appointments, and physician communication.

The pediatric doctor can work with caregivers to propose a clear delineation of tasks when it comes to IBD management tasks. The provider might suggest that the patient observe their caregivers before managing the tasks individually. While some parents may wish to take care of everything for as long as possible to reduce the burdens on their children, doctors can educate parents on the importance of helping children to be autonomous. Physicians may help their patients and families shift responsibilities by introducing the SMART Goal framework (28).

SMART goals are about turning large, lofty goals into smaller, more achievable tasks. Setting SMART goals for IBD care can allow adolescent patients and their caregivers to clarify the goals of interest, focus their attention to optimize their chances of completing the goals successfully, and ultimately, build efficacy that prepares the patient-parent dyad to take on more goals for independent disease management.

SMART is an acronym that highlights the key features of the goal:

- **S**pecific: What, where, and when will we work on this goal?
- **M**easurable: How can we evaluate if we have completed the goal or not?
- **A**chievable: Is this goal within the realm of possibility?
- **R**elevant to larger goals: Does this goal fit into my value system?
- **T**ime-sensitive: At what point will we check-in on progress to determine whether we have reached my goal?

An example of a transition-related goal may be as follows: ***“As an emerging adult patient, I want to take more ownership over managing my medications.”***

Below, we describe how to make this goal SMART:

*Specific: What, where, and when will we work on this goal?

***“I want to self-administer my Humira instead of having my parents do it for me. I want to start with my next dose which is scheduled in two weeks”***

- **M**easurable: How can we evaluate if we have completed the goal or not?

***“This goal is measurable because I will either administer the injection myself or not.”***

- **A**chievable: Is this goal within the realm of possibility?

***“Perhaps this goal will be most achievable if my parent supervises me the first time, and then I am to do it completely on my own for the next injection.”***

- **R**elevant to larger goals: Does this goal fit into my value system?

***“I feel strongly that I need to manage my own injections before I go away to college. I do not want to experience any anxiety around improper technique or not getting the medications in my system.”***

- **T**ime-sensitive: At what point will we check-in on progress to determine whether we have reached my goal?

***“We will start at my next dose with a supervised injection; after that, I will plan to administer Humira on my own.”***

This SMART framework can be implemented in relation to any disease management task. Physicians may encourage patients and families to share their goals and ask about these at patient visits. Providing support can help reinforce progress and keep young adults engaged in their efforts to manage their care.

### Master the gut-brain axis

The bi-directional relationship between gastrointestinal symptoms and psychological processes known as the gut-brain axis, emerged as a significant transition barrier in our pilot study that, to our knowledge, has not been previously recognized in transitions literature. Several patients emphasized that it can be very challenging to decipher whether their symptoms are derived from “stress” or “real” disease activity (i.e., active inflammation). Further, patients without endoscopic or histological evidence of active disease expressed feeling invalidated by physicians who implied that symptoms were “in their heads.” Optimizing education about the complex interactions of the gut and brain between may help patients to better manage their disease before transitioning to adult care. Referring patients to appropriate mental health resources, including psychological, behavioral, or psychopharmacological interventions might also be warranted for patients with more acute needs such as psychological distress and/or IBS-in-IBD (2).

Helping patients to understand the complexities of the gut-brain axis may start with education on the part of the physician. Physicians treating IBD should stay abreast of the latest research in psychogastroenterology and may access a wealth of online resources through The Rome Foundation, which aims to improve the lives of people with Disorders of Gut-Brain Interaction.

Pediatric physicians ought to talk to their patients about the relationship of stress and symptoms. Sharing videos that explain these relationships might be helpful. One example is an animated educational video provided by the University of North Carolina at Chapel Hill’s Division of Pediatric Gastroenterology that discusses functional abdominal pain: https://www.youtube.com/watch?v=65PeQyvQBHE

Simply helping patients acclimate to these patterns can help reduce stigma for patients who may feel that symptoms resulting from stress are not “real.” Physicians can validate these experiences.

They may ask:

- What do you notice about how your stress levels influence your IBD symptoms?
- Conversely, when you begin to experience the onset of IBD symptoms, how does that impact your mental state?

If patients resonate strongly with this gut-brain relationship, targeted gut-brain therapies may be of utility. It is therefore critical for doctors to identify and establish referral pathways with qualified mental health professionals in the community, such as qualified health psychologists, psychiatrists, or others whom they can refer patients to specific brain-gut or mental health therapies.

## Conclusion

With the incidence of pediatric IBD rising across the globe (32) and the high burden of IBD attributable to psychosocial factors, it is critical that these factors be addressed in preparation for transitions from pediatric to adult GI care. Notably, the findings from our pilot study were from a primarily white, privately insured cohort of medium-to-high socioeconomic status, and therefore, the interventions recommended may be of lesser value or accessibility to a more diverse cohort.

Critically, psychogastroenterology approaches to transitions may target the elimination or treatment of a particular problem (such as IBS-in-IBD or comorbid psychopathology) as well as the promotion of skills aimed at enhancing internal strengths that are relevant to gastrointestinal health (e.g., developing a disease narrative, practicing gratitude, engaging in the IBD community, setting SMART goals). While standard interventions such as those recommended in this review warrant further study, interventions should also account for patients and their families’ individual needs. Additionally, such unique approaches may be most successfully augmented by other health care professionals, in addition to gastroenterologists, to support patients in effectively transitioning and developing an adult patient identity. Ideally, such interventions can narrow the gap that adult doctors feel around particular adolescent topics and enhance the transition experience for all.

## Author contributions

MM and JF conceptualized and designed this article. Both authors drafted the initial manuscript and reviewed and revised the manuscript to completion. HK-K, LK, KG, and MD reviewed the manuscript for important intellectual and clinical content. All authors approved this article as submitted and agree to be accountable for all aspects of the work.

## Funding

The findings from our pilot study described in this article were supported by a TL1 (TR001434) grant from the National Center for Advancing Translational Sciences, National Institutes of Health. 

## Conflict of interest

MD and LK are co-founders and shareholders of Trellus Health.

The remaining authors declare that the research was conducted in the absence of any commercial or financial relationships that could be construed as a potential conflict of interest.

## Publisher’s note

All claims expressed in this article are solely those of the authors and do not necessarily represent those of their affiliated organizations, or those of the publisher, the editors and the reviewers. Any product that may be evaluated in this article, or claim that may be made by its manufacturer, is not guaranteed or endorsed by the publisher.
